# Neural markers of methylphenidate response in children with attention deficit hyperactivity disorder and the impact on executive function

**DOI:** 10.3389/fpsyt.2025.1475889

**Published:** 2025-03-13

**Authors:** Anqi Wang, Hua Yang, Yue Yang, Jie Yang, Xiaowen Yang, Qianhui Wen, Qian Wang, Hao Liu, Rong Luo

**Affiliations:** ^1^ Department of Pediatrics, West China Second University Hospital, Sichuan University, Chengdu, China; ^2^ Key Laboratory of Birth Defects and Related Diseases of Women and Children (Sichuan University), Ministry of Education, Chengdu, China

**Keywords:** attention deficit hyperactivity disorder, children, executive function, methylphenidate, p300

## Abstract

**Background:**

Attention Deficit Hyperactivity Disorder (ADHD) is a common neurodevelopmental disorder characterized by inattention, hyperactivity, and impulsivity. A core cognitive deficit in ADHD is executive function (EF) impairment, which significantly impacts daily life. Methylphenidate (MPH) is a widely used first-line treatment for ADHD, but objective biomarkers to assess treatment response are lacking. The aim of this study was to investigate the effects of MPH on executive function and identify potential neural biomarkers of response in children with ADHD using electroencephalogram (EEG).

**Methods:**

A total of 26 boys with ADHD (mean age 8.64 ± 1.30 years) participated in the study. All participants were treated with 18 mg/day of oral extended-release MPH in the morning for 8 weeks. Executive function was assessed using the BRIEF2 and Digit Span Test (DST), and event-related potentials (ERP) were measured at baseline and after 8 weeks of MPH treatment.

**Results:**

After 8 weeks of MPH treatment, significant improvements were observed in several executive function domains. BRIEF2 scores, including inhibition, self-monitoring, shifting, emotional control, initiation, working memory, planning/organization, task monitoring, and material organization, were significantly reduced (*P* < 0.05). Behavioral performance in the Go/NoGo task also improved, with shorter correct response times and higher accuracy rates (*P* = 0.002, *P* = 0.009). EEG results revealed a reduction in Nogo-P300 latency at Fz, Cz and Pz compared to baseline (*P*<0.05).

**Conclusions:**

The normalization of P300 latency following MPH treatment appears to be a reliable neural biomarker of positive treatment response in children with ADHD. MPH was associated with improvements in executive function, particularly in inhibitory control and working memory.

## 1 Introduction

Attention Deficit Hyperactivity Disorder (ADHD) is a prevalent neurodevelopmental disorder marked by persistent symptoms of inattention, hyperactivity, and impulsivity that are not consistent with a child’s developmental stage. A core cognitive deficit in ADHD is impairment in executive function (EF), which includes essential cognitive processes such as attention regulation, working memory, decision-making, impulse control, and time management (1–3). Notably, ADHD exhibits a pronounced male predominance, with a male-to-female ratio ranging from 2:1 to 3:1 in clinical populations, potentially due to differences in symptom presentation and neurobiological mechanisms (4, 5). Males with ADHD are more likely to display externalizing behaviors (e.g., hyperactivity and impulsivity) compared to females, who may exhibit internalizing symptoms, leading to underdiagnosis in females and a higher representation of males in research cohorts (6, 7). Working memory and inhibitory control are two fundamental components of EF that are interrelated. Children with ADHD often experience deficits in both areas, which contribute to inattention, academic underachievement, and difficulties in social interactions and daily tasks. These challenges frequently extend into adulthood, leading to lower high school graduation rates, decreased college enrollment, and difficulties in employment due to poor organizational skills, time management, and self-regulation (8–11). Importantly, sex differences may further modulate these outcomes; for example, males with ADHD are at higher risk for academic underachievement and conduct-related problems compared to females (12, 13). The long-lasting nature of these impairments underscores the importance of investigating interventions, such as methylphenidate (MPH), that may improve EF and, in turn, enhance academic and career opportunities for children with ADHD.

MPH is a widely used first-line treatment for ADHD, acting primarily by blocking the reuptake of dopamine (DA) and norepinephrine (NA) in the brain, which enhances their concentrations in synaptic gaps and improves EF (14). MPH is well-established for rapidly alleviating core ADHD symptoms and improving attention, hyperactivity, and impulse control, making it the preferred choice among stimulant medications (15). Studies also suggest MPH is more effective than non-stimulant treatments in improving ADHD symptoms. In China, MPH is commonly prescribed by psychiatrists to children diagnosed with ADHD, particularly for those who fail to respond to behavioral therapies alone (16). The Digit Span Test (DST) is an important indicator for assessing working memory. Karatekin & Asarnow et al. (17) have found that children with ADHD significantly underperform in the DST compared to healthy controls. Frankfort et al.’s study on cognitive function in boys with ADHD found that MPH significantly improved performance in executive function, visual memory, reaction time skills, and general cognitive abilities (18). Li Yang et al. (19) have found that MPH can improve EF in children and adolescents with ADHD, and can restore working memory to normal performance levels. Boys with ADHD demonstrate impairments in response inhibition, which can be ameliorated through the use of MPH (20, 21).Functional magnetic resonance imaging (fMRI) studies have shown that acute administration of MPH can normalize brain dysfunction in typically affected frontal areas in children with hyperactivity, potentially explaining the impact of MPH on EF (22). Patients with ADHD treated with MPH may exhibit characteristic changes in the electroencephalogram (EEG) activity (23).

In addition to its impact on dopaminergic and noradrenergic activity, there is growing evidence that MPH may also influence brain activity, particularly through alterations in event-related potentials (ERP). ERP, which provides high temporal resolution, is a valuable tool in assessing cognitive functions by measuring brain responses to stimuli in real time (24, 25). One of the most widely studied ERP components is the P300 wave, which reflects the brain’s processing of information during cognitive tasks (26, 27). Research has shown that children with ADHD have consistently lower P300 amplitudes compared to typically developing children (28). Interestingly, these differences can be improved with the use of psychostimulant medications. Lawrence et al. (29)examined the effects of MPH on boys with ADHD and observed a reduction in P300 latency following treatment. Ozdag et al. (30) found that boys with ADHD exhibited a reduction in P300 latency and an increase in amplitude following MPH treatment. Studies suggest that the P300 wave’s neural activation is linked to dopamine and other related neurotransmitters (26, 31). In Go/NoGo tasks, which test a child’s ability to respond or inhibit a response, children with ADHD show a delay in the P300 wave’s timing for both “Go” (respond) and “NoGo” (don’t respond) trials compared to healthy peers. They also exhibit reduced P300 amplitudes specifically during “NoGo” trials (32). Overall, compared to controls, children with ADHD perform worse on these tasks, and ERP analyses reveal distinct patterns of brain activity, particularly a delayed P300 latency in the “NoGo” trials (33). Interestingly, MPH treatment has been shown to normalize these P300 deficits, suggesting that ERP components could serve as reliable biomarkers for monitoring treatment efficacy in ADHD.

However, despite these promising findings, few studies have directly linked ERP components with behavioral outcomes in children during the acute phase of MPH treatment, highlighting a gap in current research. Thus, the present study aims to explore the neural markers of MPH response in children with ADHD, with a focus on EF improvements and changes in brain activity as measured by ERP. We hypothesize that MPH treatment will lead to significant improvements in both EF and ERP markers, particularly in working memory and inhibitory control, which are crucial components of EF in ADHD. Given the male predominance in ADHD and potential sex-specific neurobiological responses to stimulant medications, the current study focuses exclusively on male participants to control for confounding effects of sex differences in neural and behavioral outcomes.

## 2 Methods

### 2.1 Participants

The participants were school-aged male children (6 to 12 years old) diagnosed with ADHD at West China Second University Hospital, Sichuan University, between October 2022 and January 2024. Written informed consent was obtained from each participant’s parents, and the study was approved by the Human Research Ethics Committee of the West China Second University Hospital, Sichuan University.

In this study, all children suspected of having ADHD initially visited the pediatric neurology and psychiatry outpatient clinics at our hospital. Within one month of their first visit, they were diagnosed by two experienced pediatric neurologists and psychiatrists. Initial screening for ADHD was conducted using Conners’ Parent/Teacher Rating Scales to identify potential ADHD symptoms. Detailed clinical assessments were subsequently carried out according to Diagnostic and Statistical Manual of Mental Disorders, 5th Edition (DSM-5) standards to confirm the diagnosis of ADHD. To exclude Oppositional Defiant Disorder (ODD) and other potential psychiatric illnesses, the Kiddie Schedule for Affective Disorders and Schizophrenia for School-Age Children – Present and Lifetime Version (K-SADS-PL) was utilized. Additionally, each child underwent comprehensive medical and psychological evaluations to exclude any other variables that could affect the study’s outcomes, such as tic disorders, autism spectrum disorders, and other neurodevelopmental disorders. Participants scoring below 80 on the Wechsler Intelligence Scale for Children-Fourth Edition (WISC-IV), those who have taken any psychotropic medication (including stimulant and non-stimulant drugs) in the past three months, those who have consumed any non-psychotropic medications in the past month, and those suffering from chronic systemic diseases were all excluded from the study. The data selection process is depicted in the flowchart presented in **Supplementary Figure S1**.

In the acute phase assessment, a fixed dose of extended-release MPH was used to more effectively observe the drug’s effects and tolerability. Although there is an established framework for titrating MPH based on individual responses and clinical guidelines, we chose a standard starting dose to simplify the treatment protocol of this study. Some studies suggest that a 18 mg dose is considered a safe and effective starting dose, particularly for children beginning MPH treatment (34, 35). Therefore, in this study, participants received oral extended-release MPH treatment at a fixed dose of 18 mg every morning for 8 weeks. The effects of methylphenidate treatment were assessed in the hospital evaluation room at baseline (week 0) and after 8 weeks of treatment (36, 37).

### 2.2 Executive function assessment

The Behavior Rating Inventory of Executive Function-Parent form, Second edition(BRIEF2): It was developed by Gioia (38)et al. and assessed behavioral, affective, and cognitive abilities in executive functioning in children and adolescents aged 5-18 years. It consists of three main dimensions and nine subscales with 63 items in total. The three dimensions are as follows: (1) Behavioral Regulation Index (BRI), including inhibition and self-monitoring; (2) Emotional Regulation index (ERI), including shifting and emotional control; and (3) Cognitive regulation index (CRI), including planning/organization, organization of materials, initiation, task monitoring, and working memory. Higher scores for each factor indicated more serious behavioral problems.

Digit Span Test (DST): It is a subtest of the Wechsler Intelligence Scale for Children, Fourth Edition (WISC-IV) (39), which assesses a participant’s working memory and consists of forward digit span (FDS) and backward digit span (BDS) tasks. The longer the string of numbers the participant recited, the higher the score. DST is an important indicator for assessing working memory.

**Figure 1:**
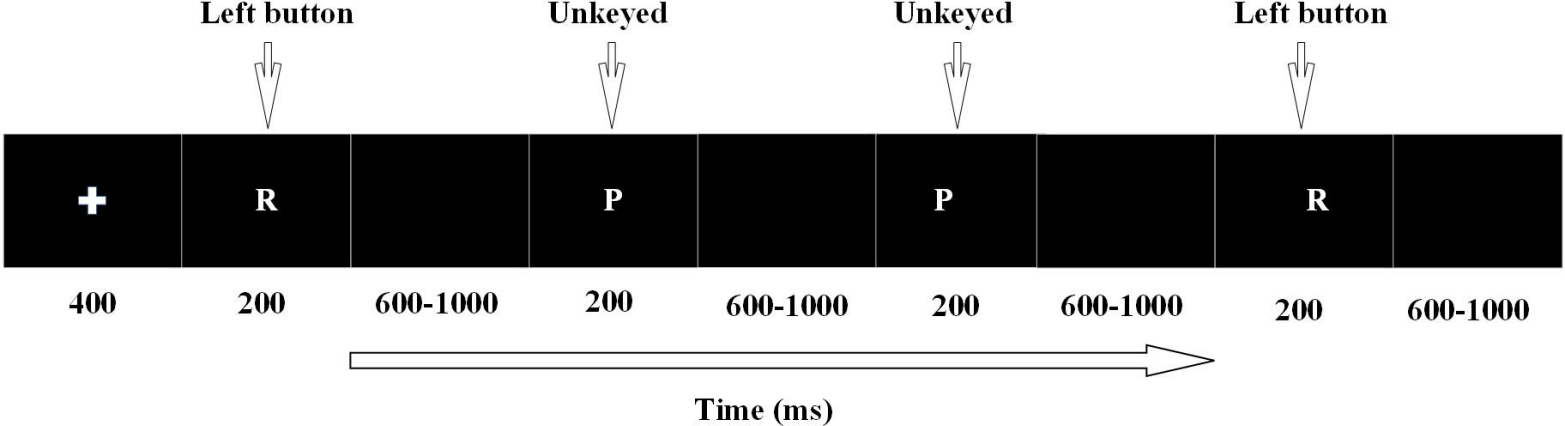
Go/Nogo task design.

Go/Nogo task: It was adapted from Serrien, at al (40). Target stimuli for the Go/Nogo task: the letter “R” as the Go stimulus and the letter “P “as the NoGo stimulus. The number of letters “R” was 144 (80%), and the number of letters “P” was 36 (20%). The task started with a cross appearing in the middle of the computer screen for 400ms as a cue, followed by the letters “R” and “P” appearing randomly in the center of the computer screen for 200ms each, with the next letter appearing after a time interval of 800 ± 200ms. Participants press the left mouse button when they see the letter “R” and do not press the button when they see the letter “P” (**Figure 1**). Variables related to behavioral performance, such as reaction time and accuracy rate, were extracted and subjected to statistical analysis. This study utilized PsychToolbox software to present visual stimuli.

### 2.3 Efficacy assessment

The Clinical Global Impressions-Improvement Scale (CGI-I) was used to classify participants with ADHD as responders or non-responders to methylphenidate (41). Scores ranged from 1 (significant improvement) to 7 (very much worse). Participants with a CGI-I score <3 were considered responders, while those with ≥3 were non-responders. The Clinical Global Impressions-Severity of Illness Scale (CGI-S) assesses illness severity on a 7-point scale: 1, no illness; 2, borderline mental illness; 3, mild illness; 4, moderate illness; 5, marked illness; 6, severe illness; and 7, most severe illness (42).

### 2.4 EEG acquisition

The participants sat in a quiet, temperature-controlled room with dim lighting to minimize external distractions during the EEG test. Nineteen Ag/AgCl electrodes were placed at Fp1, Fp2, Fz, F3, F4, F7, F8, Cz, C3, C4, Pz, P3, P4, T3, T4, T5, T6, O1, and O2 according to the International 10-20 system. The sampling rate was 2000 Hz, and the impedance between the electrodes and the scalp was kept less than 5 k Ω. Two reference electrodes (A1 and A2) were placed on both earlobes. EEG recordings while participants completed the computerized Go/Nogo task.

### 2.5 EEG processing

The procedure included downsampling to 512 Hz, 0.5 - 35 Hz bandpass filtering, averaging reference, baseline correction, artifact removal by independent component analysis (ICA), and setting the threshold to ±100 μV. Segments during responses were set with windows from −200 ms before the event to 600 ms after. The mean amplitude and latency of the P300 component were extracted from the Fz, Cz, and Pz electrodes in a 250-500 ms time window (**Table 1**).

**Table 1:** EEG processing steps.

Step	Description	Purpose
**1. Downsampling**	The original EEG data were downsampled to 512 Hz.	To reduce the amount of data without losing critical information, facilitating efficient processing.
**2. Bandpass Filtering**	A bandpass filter was applied to the data within the frequency range of 0.5 to 35 Hz.	To eliminate low-frequency drifts (e.g., movement artifacts) and high-frequency noise.
**3. Averaging Reference**	An average reference was computed by averaging the signals from all electrodes.	To minimize the effects of common noise across electrodes and enhance the detection of local brain activity.
**4. Baseline Correction**	The data were baseline corrected using the average amplitude during a pre-stimulus period (from -200 ms to 0 ms).	To remove pre-existing activity that could influence post-stimulus measurements, ensuring clearer assessments.
**5. Segmentation**	EEG data were segmented into epochs, with each epoch ranging from -200 ms before the event to 600 ms after the event.	To analyze specific time windows around events of interest.
**6. Artifact Removal**	Independent Component Analysis (ICA) was employed to identify and remove artifacts from the EEG signals.	To ensure that the analysis is based on clean EEG signals, improving the reliability of results.
**7. Threshold Setting**	A threshold of ±100 μV was applied to exclude any segments that contained excessive noise or artifacts.	To include only clean and reliable data segments in the analysis.
**8. P300 Component Extraction**	The mean amplitude and latency of the P300 component were extracted from the Fz, Cz, and Pz electrodes within the time window of 250-500 ms post-stimulus.	To evaluate the P300 component characteristics, which are indicative of cognitive processes related to stimulus evaluation.

### 2.6 Statistical analysis

The outcome metrics were analyzed using SPSS version 23.0. Data were tested for normality using the Kolmogorov-Smirnov and Shapiro-Wilk methods, with measures that conformed to normality described as “mean ± standard deviation” and those that did not conform to the normal distribution described as “median ± interquartile spacing.” Paired t-test and independent t-test were used for measures that conformed to a normal or approximately normal distribution, and nonparametric rank-sum tests were used for measures that did not conform to a normal distribution. The chi-squared test was used for between-group comparisons of categorical information.

## 3 Results

### 3.1 Demographic characteristics

The demographic characteristics of children with ADHD are presented in **Table 2**. This study enrolled 26 male children with ADHD (8.64 ± 1.30 years). Eight ADHD-inattention types (ADHD-I) (30.8%), one ADHD-hyperactive-impulsive type (ADHD-HI) (3.8%), and 17 ADHD-combined types (ADHD-C) (65.4%) were included.

**Table 2:** Demographic characteristics.

Characteristics	ADHD
N	26
Male, (%)	100
Age, mean (SD), years	8.64 ± 1.30
Subtype, No. (%)	-
ADHD-I	8 (30.8)
ADHD-HI	1 (3.8)
ADHD-C	17 (65.4)

### 3.2 Executive function

#### 3.2.1 BRIEF2 and DST

Changes in BRIEF2 after eight weeks of treatment with MPH in children with ADHD, compared to baseline, are shown in **Table 3**. Inhibition, self-monitoring, shifting, emotional control, initiation, working memory, planning/organization, task monitoring, material organization, BRI, CRI, and total scores were significantly lower (*P* < 0.05). Compared to baseline, FDS and BDS scores did not significantly change after eight weeks of MPH treatment (*P* > 0.05).

**Table 3:** Methylphenidate treatment BRIEF2 and DST change in children with ADHD.

Items	0 week	8 weeks	*P*
BRIEF2			
Inhibition	63.8077 ± 9.34674	55.1923 ± 7.31584	0.001^*^
Self-monitoring	66.6923 ± 10.01107	59.7308 ± 5.78659	0.013^*^
Shifting	59.2308 ± 7.53290	54.3462 ± 7.86355	0.014^*^
Emotional control	58.6538 ± 9.97975	53.6923 ± 6.79864	0.023^*^
Initiation	61.7308 ± 7.23081	56.6923 ± 8.69836	0.009^*^
Working memory	69.6538 ± 8.33759	64.1538 ± 7.66651	0.002^*^
Planning/Organisation	65.9615 ± 8.70164	60.1154 ± 6.43942	0.012^*^
Task monitoring	67.8846 ± 9.13160	63.2692 ± 8.79799	0.023^*^
Material organization	61.0 ± 10.38075	56.6538 ± 6.36831	0.024^*^
BRI	64.8846 ± 8.45850	57.3462 ± 6.66299	0.001^*^
ERI	59.9615 ± 8.95089	56.8846 ± 11.73142	0.223
CRI	67.0385 ± 7.52851	60.5769 ± 7.41443	0.001^*^
Total score	68.0769 ± 7.82265	63.2692 ± 7.30785	0.013^*^
DST			
FDS	7.1538 ± 1.75937	7.6154 ± 1.26734	0.103
BDS	3.8077 ± 1.49718	3.4231 ± 1.27037	0.178

BRIEF2, Behavior Rating Inventory of Executive Function-Parent form, Second edition; BRI, Behavioral Regulation Index; ERI, Emotional Regulation Index; CRI, Cognitive Regulation Index; DST, Digit Span Test; FDS, Digits Forward; BDS, Digits Backward.*P < 0.05 by Paired t-tests.

#### 3.2.2 Go/NoGo task

The results of the correctness and response time from the Go/NoGo task after MPH treatment are shown in **Table 3**. Compared to baseline, the NoGo task correct response time was significantly shorter after eight weeks of MPH treatment (*P* = 0.002). The correctness rate was also higher than baseline (*P* = 0.009) (**Table 4**).

**Table 4:** Changes in Go/NoGo task and CGI score after methylphenidate treatment.

Items	0 week	8 weeks	*P*
Go/NoGo task			
Correct response time (ms) Correctness rate (%)	611.53 ± 84.21 40.92 ± 16.36	582.57 ± 71.29 59.08 ± 16.36	0.002^a^ 0.009^a^
CGI-I score			
<3 (Mean ± SD, score) ≥ 3 (Mean ± SD, score)	- -	1.5 ± 0.53 3.06 ± 0.24	<0.001^b^ 0.001^a^
CGI-S score	4.38 ± 0.75	3.15 ± 0.83	

a by paired t – test, b by independent t – test.

### 3.3 Response to MPH

#### 3.3.1 CGI

In the study involving 26 ADHD patients, 69.2% (18/26) responded to MPH treatment (defined as CGI-I < 3), with responders having a CGI-I of 1.5 ± 0.53; 30.8% (8/26) did not respond to MPH treatment (defined as CGI-I ≥ 3), with non-responders having a CGI-I of 3.06 ± 0.24 (**Table 4**).

### 3.3.2 P300 component

**Figure 2:**
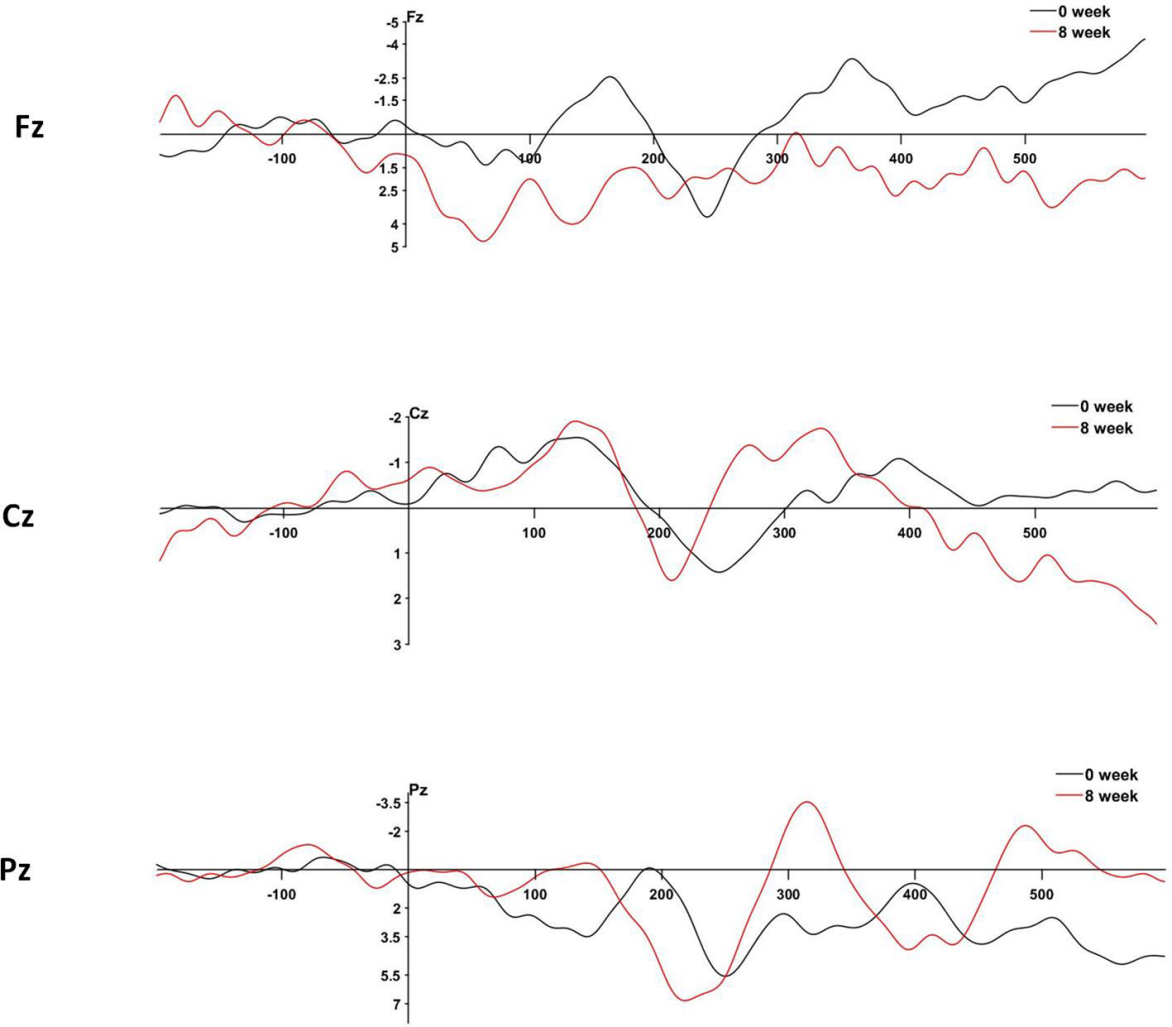
Changes in P300 composition after MPH treatment in children with ADHD. The horizontal coordinate is the time (ms), and the vertical coordinate is the amplitude of the waves (mV).

Changes in P300 components after eight weeks of treatment with MPH in children with ADHD are shown in **Table 5**. Compared to baseline, the NoGo-P300 latency at Fz was significantly reduced (*P* < 0.001). Similarly, the NoGo-P300 latency at Cz was shorter than baseline (*P* = 0.028), and the NoGo-P300 latency at Pz also decreased compared to baseline (*P* = 0.023) (**Figure 2**).

**Table 5:** Comparison of Fz, Cz, and Pz Electrode Nogo-P300 after methylphenidate treatment in Children with ADHD.

Items	0 week	8 weeks	*P*
Fz			
Latency (ms)	233.13±107.52	161.63±103.48	<0.001^*^
Amplitudes (μv)	3.39±2.66	3.63±1.85	0.693
Cz			
Latency (ms)	243.01±60.33	217.76±31.10	0.028^*^
Amplitudes (μv)	1.54±1.94	1.71±1.86	0.702
Pz			
Latency (ms)	242.11±98.32	204.81±97.92	0.023^*^
Amplitudes (μv)	5.59±4.23	6.99±3.44	0.103

*P < 0.05 by Paired t-tests.

## 4 Discussion

In this study, we explored the acute effects of MPH on EF in boys with ADHD. After 8 weeks of MPH treatment, BRIEF2 assessment results showed significant improvement in various domains of EF compared to baseline measures. Interestingly, although BRIEF2 scores significantly decreased, working memory, as assessed by DST, did not show significant changes after MPH treatment. This observation is consistent with previous studies, highlighting the discrepancy between subjective and objective cognitive function measurements in ADHD (43).These findings emphasize the complexity of evaluating treatment effects in ADHD, suggesting that a comprehensive assessment approach, combining both subjective and objective scales, is necessary to fully understand the impact of MPH on cognitive function in children with ADHD Response inhibition deficits are considered one of the core mechanisms in the pathophysiology of ADHD (44). The study by Broyd et al. (45) found that MPH significantly reduced errors in the Go/NoGo task in children with ADHD, indicating that MPH particularly improves inhibitory motor control in these children. Many studies have shown that older adolescents and young adults perform better on response inhibition tasks than children, with inhibitory processes undergoing a longer maturation process (46). Structural MRI studies have found that poor response inhibition function in ADHD patients is associated with thickening of the posterior inferior frontal cortex (47), and patients who perform poorly on the Go/NoGo task may have delayed maturation in this region. Patients with a CGI-I score of less than 3 are considered responders to MPH treatment (41). In this study, 69.2% of ADHD patients were classified as MPH responders, which is similar to the findings of Retz et al. (48), where 74.5% of patients were classified as responders. The study by Rosenau et al. (1) also showed that ADHD patients with a pre-treatment CGI-S score greater than 4, consistent with our findings, indicating significant improvement in ADHD symptoms following MPH treatment.

Previous studies have not identified objective indicators for evaluating the efficacy of MPH, and recent studies have increasingly focused on identifying biomarkers to provide precise medical care for patients with ADHD. ERP amplitudes and latency of ERP have been identified as promising biomarkers for pharmacological therapy in children with ADHD (49). The amplitude of an early subcomponent of P300 is reduced in patients with ADHD when confronted with salient or novel stimuli (50). Subcomponent P3a of the P300 is generated by the excitation of the frontal striatal nerve groups involved in the orientation and assessment of auditory or visual stimuli. Frontal striatal neural circuits are regulated by catecholamine neurotransmitters, particularly dopamine (51). Electroencephalographic studies of patients with parkinson’s disease with low dopamine levels have found a decrease in P3a amplitude (52), which increases to normal levels after the administration of stimulant medication, which is the effect of dopamine agonists (26). In this study, we found that children with ADHD who completed the Go/NoGo task after 8 weeks of treatment with MPH had elevated NoGo-P300 amplitudes and shortened latencies at Fz, Cz, and Pz. The NoGo-P300 latency was significantly shortened at Fz, Cz and Pz. Lawrence et al. (29) investigated the effects of MPH in 18 male children with ADHD and found that MPH treatment significantly shortened P300 latency, a result that is in line with our own findings. Groom et al. (53)conducted a Go/NoGo task with 28 male children with ADHD and found that methylphenidate significantly enhanced the amplitudes of error-related negativity (ERN) and error positivity (Pe). We suggest that the normalization of the MPH to the P300 component may be due to improved frontal striatal cortical network integrity and increased excitatory synaptic capacity. Our findings suggest that changes in P300 amplitude and latency may be influenced by stimulant medication, although causality cannot be definitively established in this study. Stimulant medication has been shown to persistently alter ERPs in cognitive tasks related to attention and inhibitory control (54). The simultaneous discharge of localized networks of pyramidal cells resulted in greater ERP amplitudes in the scalp, and the increase in P300 amplitude may reflect greater coordination of cortical network activation, particularly during the action execution phase of attentional processing. Stimulant drug-associated P300 alterations suggest that cognitive and behavioral deficits in patients with ADHD are associated with reduced cortical network activation and coordinated recruitment, which can be attenuated by increasing presynaptic catecholamine availability. The P300 component is by far the most promising ERP neuromarker for precision medicine in ADHD (26), and normalization of the P300 amplitude after stimulant treatment is a predictor of a positive response to such treatment.

This study found significant changes in the P300 components following MPH treatment, particularly a reduction in the Nogo-P300 latency. As an ERP marker, P300 has been shown to reflect an individual’s executive function and attention regulation during cognitive tasks. Our results suggest that MPH treatment may affect P300 components by improving cognitive control in children with ADHD. Specifically, the reduction in NoGo-P300 latency may be closely associated with MPH’s improvement of cognitive inhibition, which is consistent with findings by Pertermann et al. (55), who showed that MPH reduced neural noise in ADHD children during NoGo tasks, particularly in the theta frequency band, thus enhancing cognitive control. Moreover, the changes in the P300 components reflect improvements in executive function in ADHD children, such as faster response times and higher accuracy, indicating that P300 may serve as a potential neurobiological marker for assessing MPH treatment efficacy (23, 56). In clinical practice, the changes in P300 components provide valuable information for personalized medicine. By monitoring alterations in P300 amplitude and latency, clinicians can better assess how children with ADHD respond to MPH treatment and tailor the therapy based on each child’s neurophysiological characteristics. Therefore, P300, as a measurable biomarker, holds great potential for the development of more precise treatment strategies in the future, particularly in the personalized assessment of treatment outcomes and prediction of drug responses.

MPH is a commonly used medication for treating ADHD, and several studies have demonstrated its short-term efficacy (34, 35, 57). Our study also supports this notion, with significant improvements in children’s executive function observed after eight weeks of MPH treatment. These findings suggest that MPH can effectively enhance cognitive function in children with ADHD in the short term, particularly across multiple dimensions of executive function. However, its effectiveness may vary depending on individual differences, and the long-term efficacy still requires further validation. Unlike many studies that rely solely on behavioral rating scales, our research incorporated changes in the P300 component, providing more objective evidence for the neurobiological effects of MPH treatment.

This study has several limitations. First, it only included male children with ADHD, limiting the generalizability to females, as gender may affect treatment responses. Second, without subgroup analyses based on ADHD subtypes, the study could not assess MPH’s effects across different presentations. Finally, relying solely on the P300 component of ERPs, without incorporating other neuroimaging techniques like fMRI, limits the understanding of methylphenidate’s neural mechanisms. Future research should address these issues by including diverse samples and using multiple neuroimaging methods.

In conclusion, the normalization of the P300 component in the medial prefrontal and medial parietal regions can serve as a biological indicator of treatment efficacy in children with ADHD. This can be utilized alongside clinical measures to assist in making personalized treatment decisions for ADHD pharmacotherapy. MPH improves EF in children with ADHD, and its modulatory effects on these functions offer new insights into the underlying neurophysiological mechanisms.

## Data Availability

The original contributions presented in the study are included in the article/**Supplementary Material**. Further inquiries can be directed to the corresponding author.

## Supplementary material

Supplementary Figure 1 Flowchart of study design and data selection process.

## References

[1] Rosenau PT Openneer TJC Matthijssen AM van-de-Loo-Neus GHH Buitelaar JK van den Hoofdakker BJ. Effects of methylphenidate on executive functioning in children and adolescents with ADHD after long-term use: a randomized, placebo-controlled discontinuation study. J Child Psychol Psychiatry Allied Disciplines. (2021) 62:1444–52. doi: 10.1111/jcpp.13419 PMC9292145 33778945

[2] Harkness K Bray S Durber CM Dewey D Murias K. Assessing the contribution of measures of attention and executive function to diagnosis of ADHD or autism. J Autism Dev Disord. (2024). doi: 10.1007/s10803-024-06275-9 38478161

[3] Diamond A. Executive functions. Annu Rev Psychol. (2013) 64:135–68. doi: 10.1146/annurev-psych-113011-143750 PMC4084861 23020641

[4] Huang CL Wang JJ Ho CH. Trends in incidence rates of diagnosed attention-deficit/hyperactivity disorder (ADHD) over 12 years in Taiwan: A nationwide population-based study. Psychiatry Res. (2020) 284:112792. doi: 10.1016/j.psychres.2020.112792 31981938

[5] Ramtekkar UP Reiersen AM Todorov AA Todd RD. Sex and age differences in attention-deficit/hyperactivity disorder symptoms and diagnoses: implications for DSM-V and ICD-11. J Am Acad Child Adolesc Psychiatry. (2010) 49:217–28 e1-3. doi: 10.1097/00004583-201003000-00005 20410711 PMC3101894

[6] Merikangas KR He JP Brody D Fisher PW Bourdon K Koretz DS. Prevalence and treatment of mental disorders among US children in the 2001-2004 NHANES. Pediatrics. (2010) 125:75–81. doi: 10.1542/peds.2008-2598 20008426 PMC2938794

[7] Gershon J. A meta-analytic review of gender differences in ADHD. J Atten Disord. (2002) 5:143–54. doi: 10.1177/108705470200500302 11911007

[8] Mohamad N Rousseau KL Dowlut F Gering M Thomas KGF. Symptoms of ADHD and other common mental disorders influence academic success in South African undergraduates. J Atten Disord. (2025) 17:10870547241310659. doi: 10.1177/10870547241310659 PMC11800717 39819162

[9] Zetterqvist V Oster C Oremark A Myllys L Meyer J Ramklint M. I’ve really struggled but it does not seem to work: adolescents’ experiences of living with ADHD - a thematic analysis. BMC Psychol. (2025) 13:75. doi: 10.1186/s40359-025-02350-7 39871390 PMC11773757

[10] Smith ZR Flax M Becker SP Langberg J. Academic motivation decreases across adolescence for youth with and without attention-deficit/hyperactivity disorder: Effects of motivation on academic success. J Child Psychol Psychiatry Allied Disciplines. (2023) 64:1303–13. doi: 10.1111/jcpp.13815 37164935

[11] Sedgwick-Muller JA Muller-Sedgwick U Adamou M Catani M Champ R Gudjonsson G. University students with attention deficit hyperactivity disorder (ADHD): a consensus statement from the UK Adult ADHD Network (UKAAN). BMC Psychiatry. (2022) 22:292. doi: 10.1186/s12888-022-03898-z 35459116 PMC9027028

[12] Mahone EM Wodka EL. The neurobiological profile of girls with ADHD. Dev Disabil Res Rev. (2008) 14:276–84. doi: 10.1002/ddrr.41 PMC3534724 19072756

[13] Valera EM Brown A Biederman J Faraone SV Makris N Monuteaux MC. Sex differences in the functional neuroanatomy of working memory in adults with ADHD. Am J Psychiatry. (2010) 167:86–94. doi: 10.1176/appi.ajp.2009.09020249 19884224 PMC3777217

[14] Borycz J Pereira MF Melani A Rodrigues RJ Kofalvi A Panlilio L. Differential glutamate-dependent and glutamate-independent adenosine A1 receptor-mediated modulation of dopamine release in different striatal compartments. J Neurochem. (2007) 101:355–63. doi: 10.1111/j.1471-4159.2006.04386.x 17254024

[15] Stein MA Waldman ID Charney E Aryal S Sable C Gruber R. Dose effects and comparative effectiveness of extended release dexmethylphenidate and mixed amphetamine salts. J Child Adolesc Psychopharmacol. (2011) 21:581–8. doi: 10.1089/cap.2011.0018 PMC3243461 22136094

[16] Wang Z Wu X Yu Z Yu L. Utilization of drugs for attention-deficit hyperactivity disorder among young patients in China, 2010-2019. Front Psychiatry. (2021) 12:802489. doi: 10.3389/fpsyt.2021.802489 35222107 PMC8863856

[17] Karatekin C Asarnow RF. Working memory in childhood-onset schizophrenia and attention-deficit/hyperactivity disorder. Psychiatry Res. (1998) 80:165–76. doi: 10.1016/s0165-1781(98)00061-4 9754696

[18] Idema IME Payne JM Coghill D. Effects of methylphenidate on cognitive functions in boys with attention deficit hyperactivity disorder: Does baseline performance matter? J Consult Clin Psychol. (2021) 89:615–25. doi: 10.1037/ccp0000662 34383534

[19] Yang L Cao Q Shuai L Li H Chan RC Wang Y. Comparative study of OROS-MPH and atomoxetine on executive function improvement in ADHD: a randomized controlled trial. Int J Neuropsychopharmacol. (2012) 15:15–26. doi: 10.1017/S1461145711001490 22017969

[20] Rubia K Halari R Cubillo A Smith AB Mohammad AM Brammer M. Methylphenidate normalizes fronto-striatal underactivation during interference inhibition in medication-naive boys with attention-deficit hyperactivity disorder. Neuropsychopharmacology. (2011) 36:1575–86. doi: 10.1038/npp.2011.30 PMC3116801 21451498

[21] Vaidya CJ Austin G Kirkorian G Ridlehuber HW Desmond JE Glover GH. Selective effects of methylphenidate in attention deficit hyperactivity disorder: a functional magnetic resonance study. Proc Natl Acad Sci U S A. (1998) 95:14494–9. doi: 10.1073/pnas.95.24.14494 PMC24401 9826728

[22] Rubia K Halari R Mohammad AM Taylor E Brammer M. Methylphenidate normalizes frontocingulate underactivation during error processing in attention-deficit/hyperactivity disorder. Biol Psychiatry. (2011) 70:255–62. doi: 10.1016/j.biopsych.2011.04.018 PMC3139835 21664605

[23] Paul-Jordanov I Bechtold M Gawrilow C. Methylphenidate and if-then plans are comparable in modulating the P300 and increasing response inhibition in children with ADHD. Atten Defic Hyperact Disord. (2010) 2:115–26. doi: 10.1007/s12402-010-0028-9 21432597

[24] Javier LC Luck SJ. ERPLAB: an open-source toolbox for the analysis of event-related potentials. Front Hum Neurosci. (2014) 8:213–. doi: 10.3389/fnhum.2014.00213 PMC3995046 24782741

[25] Epp T Skrenes A Chao T Krigolson OE Schutz CG. Associations of the P300 event-related potentials and self-reported craving in substance use disorders: A systematic review. Eur Addict Res. (2023) 29:406–16. doi: 10.1159/000533147 37820586

[26] Peisch V Rutter T Wilkinson CL Arnett AB. Sensory processing and P300 event-related potential correlates of stimulant response in children with attention-deficit/hyperactivity disorder: A critical review. Clin Neurophysiol. (2021) 132:953–66. doi: 10.1016/j.clinph.2021.01.015 PMC7981253 33677205

[27] Polich J. Updating P300: an integrative theory of P3a and P3b. Clin Neurophysiol. (2007) 118:2128–48. doi: 10.1016/j.clinph.2007.04.019 PMC2715154 17573239

[28] Kaiser A Aggensteiner PM Baumeister S Holz NE Banaschewski T Brandeis D. Earlier versus later cognitive event-related potentials (ERPs) in attention-deficit/hyperactivity disorder (ADHD): A meta-analysis. Neurosci Biobehav Rev. (2020) 112:117–34. doi: 10.1016/j.neubiorev.2020.01.019 31991190

[29] Lawrence CA Barry RJ Clarke AR Johnstone SJ McCarthy R Selikowitz M. Methylphenidate effects in attention deficit/hyperactivity disorder: electrodermal and ERP measures during a continuous performance task. Psychopharmacol (Berl). (2005) 183:81–91. doi: 10.1007/s00213-005-0144-y 16160877

[30] Ozdag MF Yorbik O Ulas UH Hamamcioglu K Vural O. Effect of methylphenidate on auditory event related potential in boys with attention deficit hyperactivity disorder. Int J Pediatr Otorhinolaryngol. (2004) 68:1267–72. doi: 10.1016/j.ijporl.2004.04.023 15364497

[31] Arnett AB Flaherty BP. A framework for characterizing heterogeneity in neurodevelopmental data using latent profile analysis in a sample of children with ADHD. J Neurodev Disord. (2022) 14:45. doi: 10.1186/s11689-022-09454-w 35922762 PMC9351075

[32] Morand-Beaulieu S Lavoie ME. Cognitive and motor event-related potentials in Tourette syndrome and tic disorders: A systematic review. Clin Neurophysiol. (2019) 130:1041–57. doi: 10.1016/j.clinph.2018.10.022 30578044

[33] Morand-Beaulieu S Smith SD Ibrahim K Wu J Leckman JF Crowley MJ. Electrophysiological signatures of inhibitory control in children with Tourette syndrome and attention-deficit/hyperactivity disorder. Cortex. (2022) 147:157–68. doi: 10.1016/j.cortex.2021.12.006 PMC8816877 35042055

[34] Luo X Dang C Guo J Li D Wang E Zhu Y. Overactivated contextual visual perception and response to a single dose of methylphenidate in children with ADHD. Eur Arch Psychiatry Clin Neurosci. (2024) 274:35–44. doi: 10.1007/s00406-023-01559-0 36725736

[35] Xu Y Chung H Shu M Liu Y Zhang Y Qiu H. Dose titration of osmotic release oral system methylphenidate in children and adolescents with attention-deficit hyperactivity disorder: a retrospective cohort study. BMC Pediatr. (2023) 23:38. doi: 10.1186/s12887-023-03850-4 36683085 PMC9869580

[36] Coghill D Seth S. Effective management of attention-deficit/hyperactivity disorder (ADHD) through structured re-assessment: the Dundee ADHD Clinical Care Pathway. Child Adolesc Psychiatry Ment Health. (2015) 9:52. doi: 10.1186/s13034-015-0083-2 26587055 PMC4652349

[37] Tamminga HGH Reneman L Schrantee A Bottelier MA Bouziane C Geurts HM. Do effects of methylphenidate on cognitive performance last beyond treatment? A randomized placebo-controlled trial in boys and men with ADHD. Eur Neuropsychopharmacol. (2021) 46:1–13. doi: 10.1016/j.euroneuro.2021.02.002 33735707

[38] Gioia GA Isquith PK Guy SC Kenworthy L. BRIEF-2: behavior rating inventory of executive function: professional manual. Lutz, FL: Psychological Assessment Resources. (2015). doi: 10.1037/t79467-000

[39] Baron IS. Test review: Wechsler intelligence scale for children-fourth edition (WISC-IV). Child Neuropsychol. (2005) 11:471–5. doi: 10.1080/09297040590951587 16306021

[40] Serrien DJ Orth M Evans AH Lees AJ Brown P. Motor inhibition in patients with Gilles de la Tourette syndrome: functional activation patterns as revealed by EEG coherence. Brain. (2005) 128:116–25. doi: 10.1093/brain/awh318 15496435

[41] Arnett AB Rutter TM Stein MA. Neural markers of methylphenidate response in children with attention deficit hyperactivity disorder. Front Behav Neurosci. (2022) 16:887622. doi: 10.3389/fnbeh.2022.887622 35600991 PMC9121006

[42] Matthijssen AM Dietrich A Bierens M Kleine Deters R van-de-Loo-Neus GHH van den Hoofdakker BJ. Continued benefits of methylphenidate in ADHD after 2 years in clinical practice: A randomized placebo-controlled discontinuation study. Am J Psychiatry. (2019) 176:754–62. doi: 10.1176/appi.ajp.2019.18111296 31109200

[43] Hai T Duffy HA Lemay JA Lemay JF. Impact of stimulant medication on behaviour and executive functions in children with attention-deficit/hyperactivity disorder. World J Clin Pediatr. (2022) 11:48–60. doi: 10.5409/wjcp.v11.i1.48 35096546 PMC8771318

[44] Usai MC. Inhibitory abilities in girls and boys: More similarities or differences? J Neurosci Res. (2023) 101:689–703. doi: 10.1002/jnr.25034 35266196

[45] Broyd SJ Johnstone SJ Barry RJ Clarke AR McCarthy R Selikowitz M. The effect of methylphenidate on response inhibition and the event-related potential of children with attention deficit/hyperactivity disorder. Int J Psychophysiol. (2005) 58:47–58. doi: 10.1016/j.ijpsycho.2005.03.008 15925419

[46] Johnstone SJ Dimoska A Smith JL Barry RJ Pleffer CB Chiswick D. The development of stop-signal and Go/Nogo response inhibition in children aged 7-12 years: performance and event-related potential indices. Int J Psychophysiol. (2007) 63:25–38. doi: 10.1016/j.ijpsycho.2006.07.001 16919346

[47] Zhan C Liu Y Wu K Gao Y Li X. Structural and functional abnormalities in children with attention-deficit/hyperactivity disorder: A focus on subgenual anterior cingulate cortex. Brain Connect. (2017) 7:106–14. doi: 10.1089/brain.2016.0444 PMC5359690 28173729

[48] Retz W Rosler M Fischer R Ose C Ammer R. Methylphenidate treatment of adult ADHD patients improves the degree of ADHD severity under routine conditions. J Neural Transm (Vienna). (2020) 127:1427–34. doi: 10.1007/s00702-020-02226-7 PMC7497302 32880706

[49] Konopka LM Zimmerman EM. Neurofeedback and psychopharmacology: designing effective treatment based on cognitive and EEG effects of medications. Clin Neurotherapy. (2014), 55–84. doi: 10.1016/B978-0-12-396988-0.00003-9

[50] Barry RJ Johnstone SJ Clarke AR. A review of electrophysiology in attention-deficit/hyperactivity disorder: II. Event-related potentials. Clin Neurophysiol. (2003) 114:184–98. doi: 10.1016/s1388-2457(02)00363-2 12559225

[51] Chu CL Lee IH Chi MH Chen KC Chen PS Yao WJ. Availability of dopamine transporters and auditory P300 abnormalities in adults with attention-deficit hyperactivity disorder: preliminary results. CNS Spectr. (2018) 23:264–70. doi: 10.1017/S1092852917000049 28847342

[52] Solis-Vivanco R Rodriguez-Violante M Rodriguez-Agudelo Y Schilmann A Rodriguez-Ortiz U Ricardo-Garcell J. The P3a wave: A reliable neurophysiological measure of Parkinson’s disease duration and severity. Clin Neurophysiol. (2015) 126:2142–9. doi: 10.1016/j.clinph.2014.12.024 25655938

[53] Groom MJ Liddle EB Scerif G Liddle PF Batty MJ Liotti M. Motivational incentives and methylphenidate enhance electrophysiological correlates of error monitoring in children with attention deficit/hyperactivity disorder. J Child Psychol Psychiatry Allied Disciplines. (2013) 54:836–45. doi: 10.1111/jcpp.12069 PMC3807603 23662815

[54] Hawk LW Jr. Fosco WD Colder CR Waxmonsky JG Pelham WE Jr. Rosch KS. How do stimulant treatments for ADHD work? Evidence for mediation by improved cognition. J Child Psychol Psychiatry Allied Disciplines. (2018) 59:1271–81. doi: 10.1111/jcpp.12917 PMC10043810 29733106

[55] Pertermann M Bluschke A Roessner V Beste C. The modulation of neural noise underlies the effectiveness of methylphenidate treatment in attention-deficit/hyperactivity disorder. Biol Psychiatry Cognit Neurosci Neuroimaging. (2019) 4:743–50. doi: 10.1016/j.bpsc.2019.03.011 31103546

[56] Roca P Mulas F Gandia R Ortiz-Sanchez P Abad L. Executive functioning and evoked potentials P300 pre- and post- treatment in attention deficit hyperactivity disorder. Rev Neurol. (2013) 56 Suppl 1:S107–18. 23446712

[57] An L Cao XH Cao QJ Sun L Yang L Zou QH. Methylphenidate normalizes resting-state brain dysfunction in boys with attention deficit hyperactivity disorder. Neuropsychopharmacology. (2013) 38:1287–95. doi: 10.1038/npp.2013.27 PMC3656372 23340519

