# Supplementary figures and images for: Neural markers of methylphenidate response in children with attention deficit hyperactivity disorder and the impact on executive function
Source: Front Psychiatry. 2025 Mar 13;16:1475889. doi: 10.3389/fpsyt.2025.1475889 (PMC11966463; doi:10.3389/fpsyt.2025.1475889)

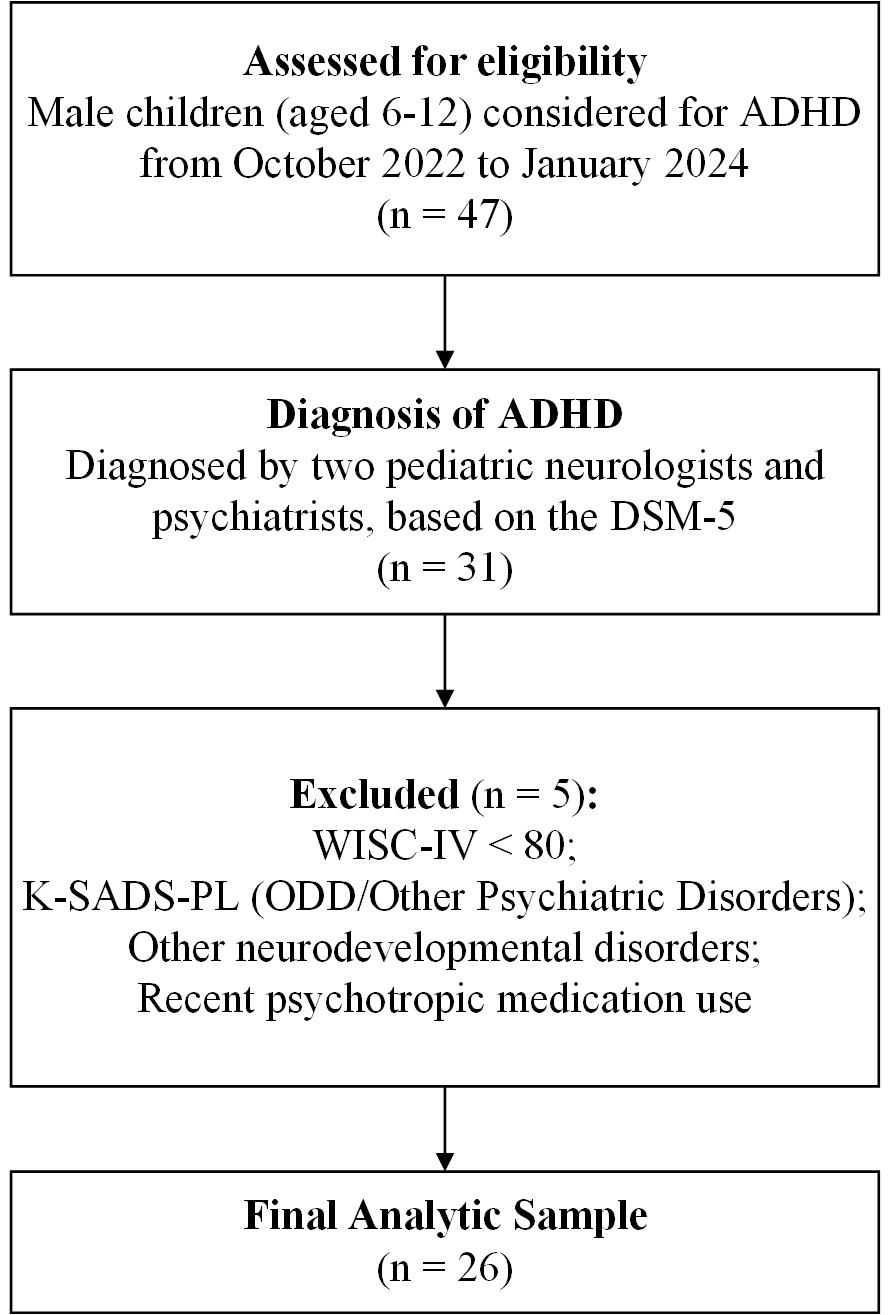

Supplement: Supplementary Figure 1 — Flowchart of study design and data selection process. [file Image1.jpeg]
